# Domestication‐Admixed Atlantic Salmon ( *Salmo salar* ) Establish a Productive Population in the Wild

**DOI:** 10.1111/ele.70319

**Published:** 2026-02-13

**Authors:** Alison C. Harvey, Øystein Skaala, Francois Besnier, Britt Iren Østebø, Anne Grete Sørvik, Per Tommy Fjeldheim, Laila Unneland, Marine S. O. Brieuc, Fernando Ayllon, Kjell R. Utne, Monica F. Solberg, Kevin A. Glover

**Affiliations:** ^1^ Institute of Marine Research Bergen Norway

**Keywords:** admixture, Atlantic salmon, colonisation, domestication, introgression, microsatellites, natural selection, productivity, SNPs, strayers

## Abstract

Widespread aquaculture escapes have led to domestication‐admixture in many wild Atlantic salmon populations, widely regarded as a threat to their evolutionary trajectory and persistence amid historically low population numbers. Although decades of research document reduced fitness of domesticated‐admixed offspring in the wild, productivity measurements of domestication‐admixed or feral salmon populations are lacking. Over a 10‐year period, we document colonisation of a river by highly (average 37%) domestication‐admixed salmon using up‐ and downstream traps, genomic data and genetic identification of over 4000 spawners and smolts. Colonisers were identified as strays originating from admixed neighbouring rivers. The resulting population now displays freshwater and marine productivity within ranges observed in wild populations. Our data therefore demonstrate that domestication‐admixed individuals can rapidly establish populations in the wild, likely facilitated in this case by an absence of local competition. Furthermore, high levels of domestication admixture do not preclude a productive population trajectory.

## Graphical abstract

We document colonisation of a river by highly (average 37%) domestication‐admixed salmon using genomic and phenotypic data. The resulting population now displays freshwater and marine productivity within ranges observed in wild populations. Our data demonstrate that domestication‐admixed individuals can rapidly establish populations in the wild, likely facilitated in this case by an absence of local competition, and further that high levels of domestication admixture do not preclude a productive population trajectory.

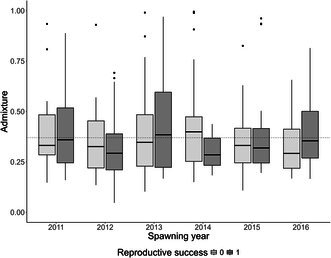

## 1 Introduction

The domestication of plants and animals is one of the most important steps in the evolution of humanity (Diamond 2002). Over time, humans have altered the genotype and phenotype of numerous species away from their wild conspecifics by both direct and indirect selection for human‐desired traits. Within captivity, domesticated organisms display higher productivity than their wild conspecifics due to human facilitation, while in the wild, the effects of captive breeding can cause a rapid fitness decline (Araki et al. 2007; Farquharson et al. 2018). Therefore, interbreeding between wild and domesticated conspecifics is considered to be detrimental to wild populations as it could erode genetic integrity and potentially increase the risk of extinction. There is, however, still limited understanding of how interbreeding with domesticated genes, referred to here as admixture, affects long‐term fitness and productivity in nature.

Studies of the long‐term consequences of domestication‐admixture in the natural environment are rare due to the time, expense, and infrastructure needed to accomplish them. A major proportion of the research into how domestication affects wild conspecifics stems from studies in fish, and in particular, the Atlantic salmon ( *Salmo salar* L.). Atlantic salmon are anadromous, spending their early life (1–6 years) in freshwater before leaving rivers as smolts, where they migrate to the ocean to feed and grow (Webb et al. 2007). After a variable number of years (1–5 years) in the ocean, Atlantic salmon return to their natal rivers to spawn (for a lifecycle overview see Figure S1) (Klemetsen et al. 2003). The fidelity of wild Atlantic salmon to their natal rivers can generate local adaptations to their river environment (Fraser et al. 2011). Salmon do not always return to their natal river, and straying, where fish originating from one river enter another river to spawn, is estimated to occur in 3%–6% of a population (Thorstad et al. 2011), however, the incidence of straying is higher in hatchery‐reared salmon (Jonsson et al. 2003). Straying may also contribute to recolonization of previously utilised habitats within the Atlantic salmon's native range (Milner et al. 2008; Griffiths et al. 2011).

The number of Atlantic salmon farmed in open net pens throughout the world now eclipses the reported numbers of wild conspecifics by a factor of 500 (ICES 2024), with wild Atlantic salmon red‐listed by IUCN (Darwall 2023). Escapes from aquaculture facilities are frequent, and as a result, genetic interactions between domesticated and wild salmon occur on a global scale, with well‐documented incidences of admixture between conspecifics in almost every region in the north Atlantic where salmon are commercially farmed and wild salmon reside (Gudmundsson et al. 2013; Gilbey et al. 2021; Diserud et al. 2023; San Román et al. 2025).

Studies have shown that directional selection for aquaculture‐desired traits has resulted in the offspring of domesticated salmon displaying lower survival relative to the offspring of wild conspecifics during the full or parts of the life cycle under natural experimental conditions (Fleming et al. 2000; McGinnity et al. 2003; Skaala et al. 2012). Similarly, studies have shown genetic‐based phenotypic changes in wild populations that have been invaded by escaped domesticated salmon (Bolstad et al. 2017; Bolstad et al. 2021, Besnier, Skaala, et al. 2022), including a potential homogenisation effect of domestication‐admixture (Skaala et al. 2006; Glover et al. 2012), whereby local adaptations and/or genetic diversity among populations could be eroded over time. Therefore, admixture from escaped domesticated salmon is widely accepted as a threat to the genetic integrity, fitness and long‐term evolutionary trajectory of wild salmon populations (Hindar et al. 1991; Laikre et al. 2010; Glover et al. 2017). However, while interactions between wild and domesticated Atlantic salmon may be regarded as the most extensively studied interaction of this kind across all taxa, thus far, very few studies have directly quantified the freshwater and marine productivity of a non‐experimental domestication‐admixed population in the natural environment (Kinnison et al. 2008).

Here, we aimed to understand to what degree domestication admixture influences population productivity in the natural environment using individual‐level admixture estimates from Atlantic salmon spawners that have recently colonised a river with no local salmon population, together with genetic pedigree analyses of out‐migrating smolts from the same river. We first examined whether individual admixture had an effect on reproductive success by comparing admixture levels of spawners with and without offspring that survived the initial fresh‐water phase of the life cycle. Furthermore, we examined how individual admixture influenced the number of offspring assigned to a spawner. We then compared the marine and freshwater productivity in our admixed population to existing wild populations from rivers in Norway and Scotland.

## 2 Materials and Methods

### 2.1 The Study Area and Sampling

**FIGURE 1:**
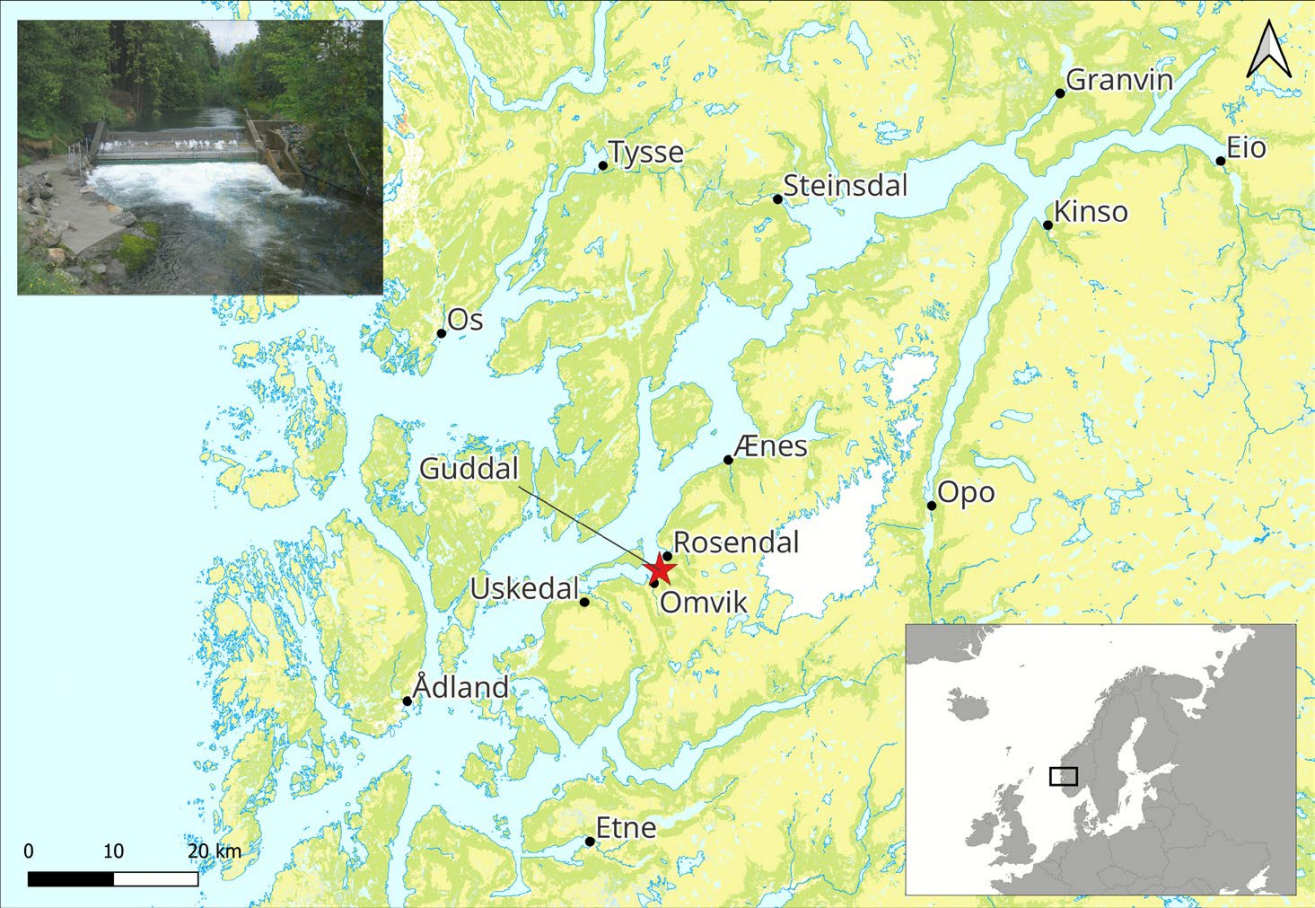
The location of the river Guddal (red star) and the rivers in the genetic baseline used to assign the spawners to potential river of origin in the Hardangerfjord in western Norway, and (upper inset) the smolt trap in the river.

The river Guddal is located in the Hardangerfjord, western Norway (Figure 1). The partially glacier‐fed river has a total length of approximately 13.5 km where the lowermost 4 km is accessible for anadromous salmonids. The river supports a population of anadromous brown trout ( *Salmo trutta* L.) and had sporadic catches of salmon reported between 1968 and 2008 (Skurdal et al. 2001; Sægrov and Urdal 2012; Skaala et al. 2019). The reason for the lack of a stable salmon population in the river may be linked to the general decline in Atlantic salmon combined with potential overharvesting and/or underreporting of angling catches of a naturally small population. Between 2000 and 2021, a Wolf trap to capture out‐migrating smolts and a spawner trap to capture ascending spawners were continually operated by the Institute of Marine Research (IMR). Between 2003 and 2011, IMR conducted two studies investigating the relative performance of domesticated, hybrid and wild Atlantic salmon planted in the river as fertilised eggs and/or smolts, with all experimental fish returning as adults to the river removed upon re‐entry (Skaala et al. 2012; Skaala et al. 2019). Throughout this period, ascending fish and smolt migration were monitored closely and the river was essentially closed to any up‐migrating salmon. In 2011 and onwards, after the above experimental work had ceased, phenotypically wild salmon spawners (i.e., not visibly an escaped farmed salmon or a salmon produced in the previous experiments, as assessed by outward characteristics and scanning for external and internal tags) were allowed to enter the river each year. All salmon and trout spawners entering the upstream trap during the years 2011–2021 (the colonisation period studied here) were anesthetised and sampled for size, phenotypic sex and a small tissue sample was taken. Fish that were classified as wild by scale reading and external phenotype were thereafter released to continue their spawning migration. For extensive details of the field station and traps see Skaala (2017).

**FIGURE 2:**
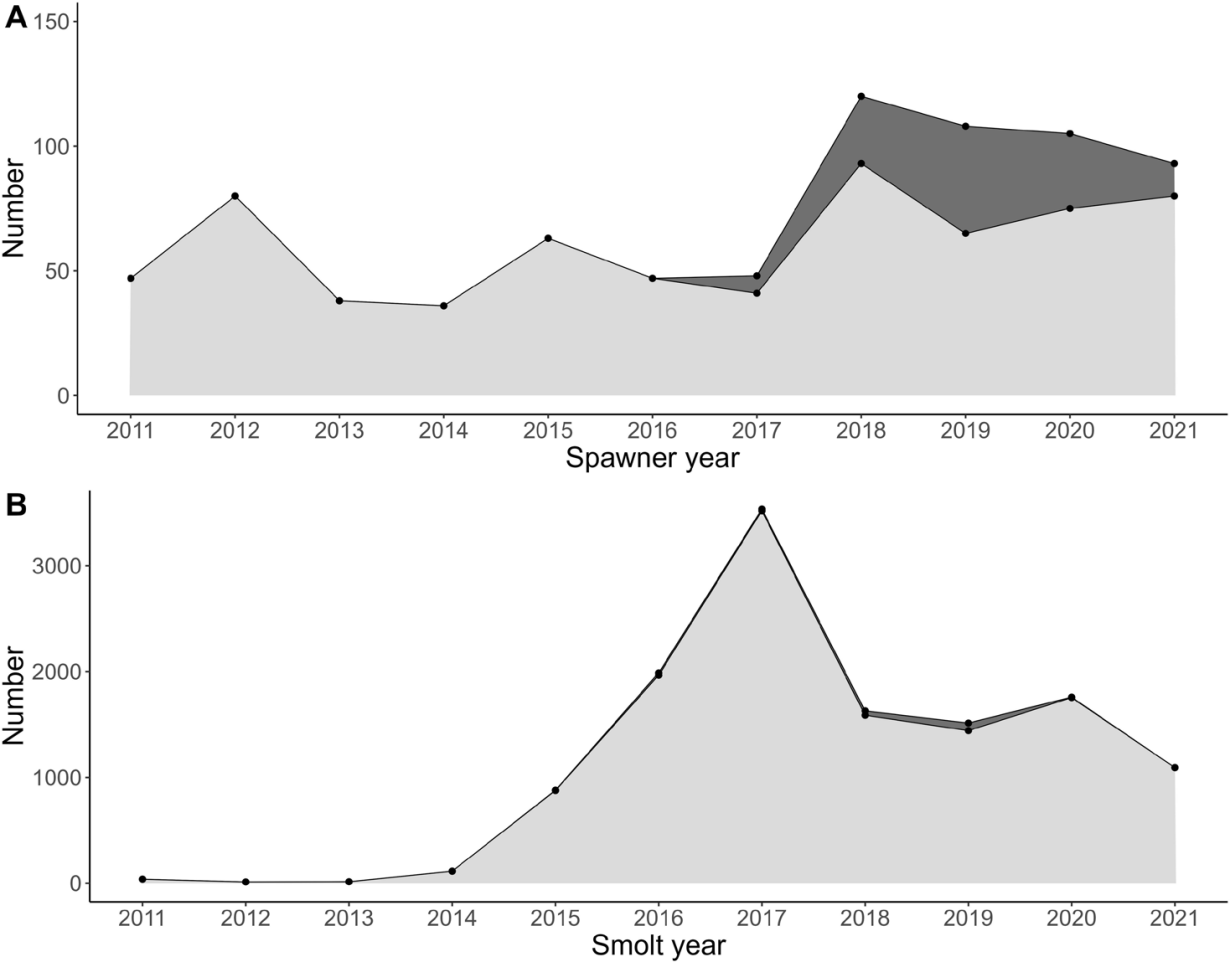
(A) The numbers of spawners of both sexes entering the trap each year, and (B) the number of smolts leaving the river each year from 2011 to 2021. The number of smolts that we identified using genetic methods as returning spawners is depicted by the dark grey areas in panel A and B; these numbers have been tripled to depict the estimated total number as only one third of the smolts leaving the river were genotyped.

The numbers of salmon entering in 2011 and subsequent years were higher than historically observed for the river (Figure S2), which is likely the result of the higher marine survival seen in most rivers located in western Norway during that time period. After the initial colonisation by these adults, a marked increase was observed in the number of salmon smolts leaving the river, with between 113 (2014) and 3514 (2017) smolts leaving each year between 2014 and 2021 (Figure 2). In 2016, approximately 75,000 eyed eggs originating from broodstock from a nearby river in the Hardangerfjord (the river Etne) were planted out in the river Guddal in collaboration with river owners and local management authorities to supplement the population after the experiments in 2003–2011 were terminated. The parental broodstock were fin‐clipped and genotyped at IMR using a panel of 16 microsatellite markers (Glover et al. 2020), therefore, we were able to genetically assign any smolts originating from those plantings back to family. All fish resulting from these plantings were removed from the entire dataset and have thus not contributed to any survival nor productivity measurements.

Over the years 2011–2021, 822 phenotypically wild Atlantic salmon entered the river, of which 785 were successfully genotyped with 30 microsatellite loci including SDY to determine their sex (as in Harvey et al. 2019) for parentage and pedigree reconstruction. Through the years 2014–2021, 12,503 wild Atlantic salmon smolts left the river and were sampled, and approximately one third were genotyped in the present study. Of those 3760 smolts subsampled from 2014 to 2021, 192 fish were removed for missing genetic or phenotypic data, including two genetic duplicates, leaving 3508 fish in the smolt dataset.

### 2.2 Admixture Estimates

All 291 adults entering Guddal in 2011–2016 were analysed with 50,000 genome‐wide SNPs (as in Besnier, Ayllon, et al. 2022) to estimate their admixture with domesticated Atlantic salmon. When using spawners between 2011 and 2016, the full range of potential descendants from these spawning years is within the out‐migrating smolt samples up to 2021. Any fish that spawned in 2017 or later may produce smolts that would leave the river later than 2021 and thus fall out of our range of available smolt samples.

The degree of domestication‐admixture of the adult salmon entering Guddal was computed using a reference material of domesticated salmon escapees captured in the nearby river Etne between 1989 and 2012 (*N* = 211), and historical samples of wild salmon from the nearby rivers Etne (1983 and 1984; *N* = 82), Eio (1988, 1990, 1994, and 1995; *N* = 31) and Granvin (1990–1996; *N* = 22). The admixture level of each Guddal spawner was evaluated using the 3172 SNPs providing the best separation between the domesticated and wild reference material using the software STRUCTURE as described in Besnier, Ayllon, et al. (2022), Besnier, Skaala, et al. (2022) and Karlsson et al. (2016).

### 2.3 Colonisation and Recruitment

As the salmon entering Guddal were likely strayers originating from rivers in the surrounding area, a genetic mixture analysis was performed using the *rubias* package (Moran and Anderson 2019) in R (R Core Team 2024) where each spawner was compared to a baseline of samples from rivers in the Hardangerfjord (see (Harvey et al. 2019) for details on the baseline rivers) and their probability of belonging to each river is calculated using Bayesian inference. Only those fish assigned with a probability of more than 0.80 were taken to be correctly assigned. The *rubias* package was also used to detect genetic duplicates within a year (i.e., a fish that entered/exited the river more than once in the same year) and between years (potential repeat spawner or smolt returning to the river to spawn as a recruit to the population).

### 2.4 Freshwater and Marine Productivity Analyses

The number of smolts produced per unit area, smolt density, is often used to quantify freshwater productivity (Saegrov et al. 2001). However, accurate estimation of smolt production in rivers without trapping facilities such as in the river Guddal is difficult and subject to methodological and sampling challenges. To estimate freshwater productivity, we calculated smolt production using the 4 km section of the river available to anadromous fish (41,578 m^2^) (Personal communication B. Skår, NORCE). The average number of smolts leaving the river between 2015 and 2021 was divided by the area to estimate smolt production per 100 m^2^. Marine survival was estimated by dividing the number of smolts genetically identified as returning spawners by the total number of genotyped smolts. The overall marine survival was estimated by using all subsampled smolts from the 2015 to 2019 smolt year classes, as there were no returning smolts detected for the 2014 or 2021 smolt year classes (2021 smolt year class would return outside the period of this study). Marine survival for each smolt year class was then also calculated as above to investigate variation among the year classes. Marine survival for the 2020 smolt year class would be underestimated as not all smolts return as one sea winter spawners, and thus smolts returning as older spawners would return outside the period for this study.

### 2.5 Reproductive Success

The software Colony Version 2.0.6.8 (Jones and Wang 2010) was used to infer familial relationships between spawners and smolts (i.e., parent‐offspring). The parentage analysis was conducted for each spawning year separately (2011–2016). The analyses were conducted assuming both male and female polygamy and possible inbreeding. The combined full‐likelihood and pair‐likelihood score method was used with a very high likelihood precision and a medium run length with updated allele frequencies.

All statistics were conducted using the R software version 4.3.1 (R Core Team 2024). We used generalised linear models to investigate, firstly, how domestication‐admixture influenced reproductive success of the adults from 2011 to 2016. We define reproductive success here as the genetic assignment of a spawner as a parent to one or more of the subsampled smolts, that is, did the adult produce smolt or not. Individual adult reproductive success was modelled as a binary response: 1 = assigned as a parent; 0 = not assigned as a parent and fitted using a generalised linear mixed model. Individual admixture (proportion between 0 and 1) was modelled as a continuous explanatory variable, and sex (2 levels) was modelled as a categorical explanatory variable. Size and sea age were correlated, and size and admixture were correlated (see below, where the relationship between size and admixture is investigated further); therefore, size and sea age were omitted from the models. The interaction term was admixture and sex. Year was modelled as a random intercept variable.

(1.1) Reproductive success~Admixture+Sex+Admixture:Sex

Secondly, we investigated how individual admixture levels influence the number of smolts produced by a successfully assigned parental spawner, that is, using only those spawners assigned as parents. One individual with 378 assigned offspring was removed from the dataset to improve model fit. The individual was not an erroneous result; rather, it was removed due to its influence on the residuals. The number of offspring produced per spawner was modelled using a generalised linear model with a negative binomial distribution. Model variables were as above for the model of reproductive success (model 1.1), apart from that year was modelled as a fixed categorical explanatory variable as this model fit better than one with year as a random intercept variable. We present the data in Figure S4 per year to show the variability between the years.

We used a generalised linear model with a Gamma distribution to model the effect of individual admixture and sex on the size (length, cm) of the spawners. Size (length in cm) was the continuous response variable, and sex (2 levels) was the categorical explanatory variable. Individual admixture (proportion between 0 and 1) was modelled as a continuous explanatory variable. The interaction term was admixture and sex. As sea age was correlated with size, it was not included in the model.

(1.2) Size~Admixture+Sex+Admixture:Sex

All models were modelled using the *glmmTMB* package (Brooks et al. 2017). The drop1 function was used to evaluate the significance of the covariates in the model by ranking nested models using the AIC criterion; differences between levels of significant categorical explanatory variables or interaction terms were investigated with simple slopes analysis from the *interactions* package (Long 2024) where applicable, and model fit was assessed using the *DHARMa* package (Hartig 2022).

Finally, we used a two‐sample *t* test to investigate whether the average admixture estimate of the spawners was significantly different from the average admixture of the offspring with both assigned parents (where the offspring admixture was estimated as the average between the parents' admixture) that were recruited back to the population.

## 3 Results

### 3.1 Colonisation Timeline and Self‐Recruitment

The first wild spawners were permitted to enter the river to spawn in 2011 (Figures 2 and S2). After the initial influx, smolt production rapidly increased from 2015 onwards, with the number of smolts leaving the river increasing from a handful to almost 1000 in 2015 (Figure 2). We used an existing genetic baseline of all salmon rivers in the region to assign the river origin of the spawners straying into the river Guddal in the initial colonisation period 2011–2016. Most of the fish entering the river Guddal were assigned to the nearby rivers Uskedal (51%) or Etne (32%) (Figure S3), both of which are the largest rivers located in the middle and outer parts of the Hardangerfjord, respectively (Figure 1).

Through individual DNA profiling to check for genetic duplicates across the years, we identified 35 smolts leaving the river from 2015 to 2020 that returned to the river as spawners from 2017 and onwards (Figure 2B,A, respectively).

### 3.2 Productivity in the River

The anadromous section of the river Guddal consists of a total estimated area of 41,578 m^2^. From the average annual number of 1662 smolts migrating out of the river in the period 2015 to 2021 divided by the total estimated area, we computed the annual average freshwater productivity to be ~4 (2–9) smolts per 100 m^2^ (Table S1). The egg‐planting that took place in the river in 2016 described in the methods resulted in approximately 750 smolts over the years 2017–2020. All of these were removed from the smolt productivity estimate but would have increased the estimate of smolt density per 100 m^2^ slightly.

**FIGURE 3:**
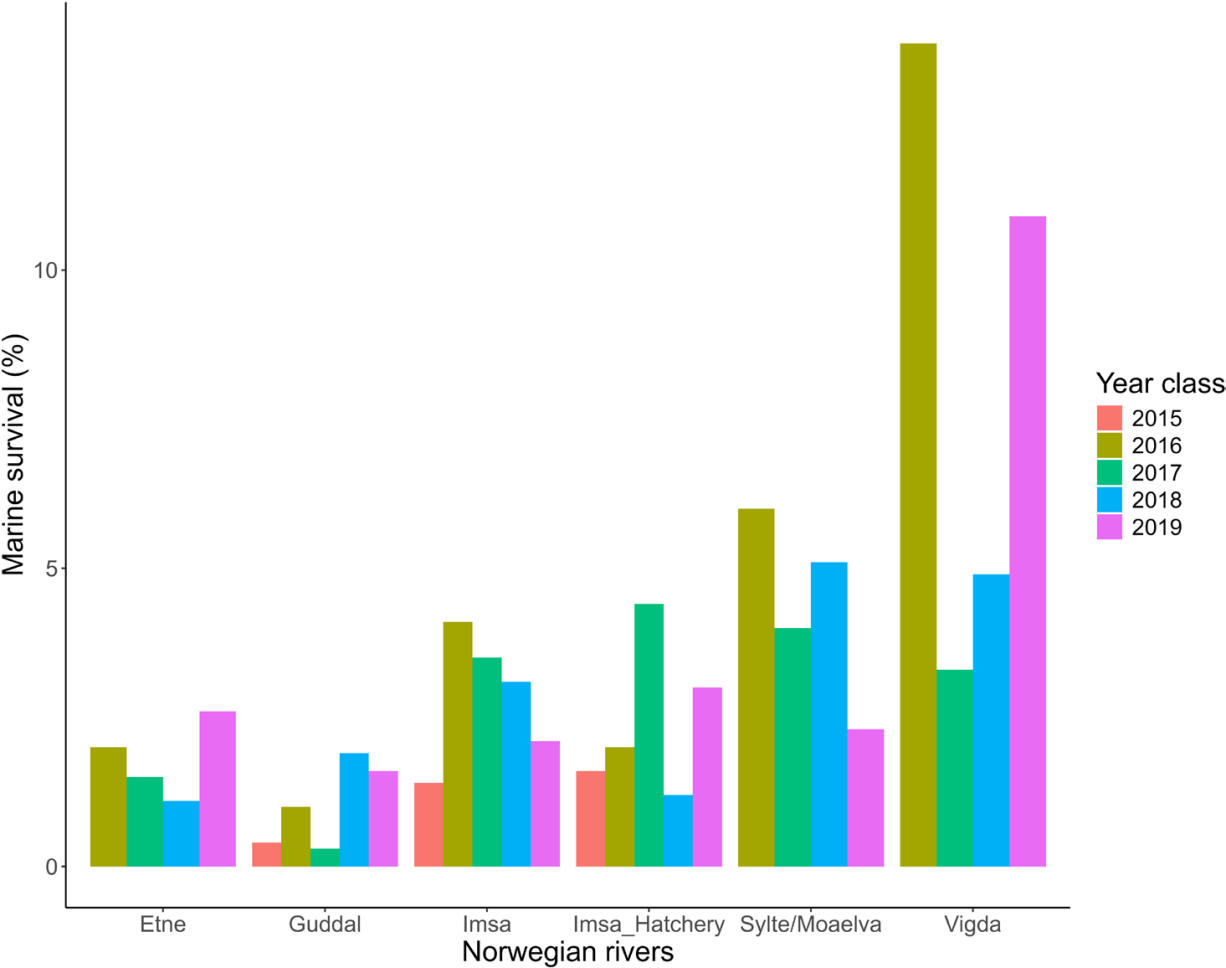
Marine survival of the river Guddal for smolt years 2015–2019 compared to other Norwegian rivers with official reporting of marine survival for wild and hatchery salmon for the same smolt year classes. The marine survival estimates were taken from ICES (60). Note that these estimates have been aggregated from 1SW and 2SW survival estimates to show overall survival.

We successfully genotyped 2826 out‐migrating smolts between 2015 and 2019, equating to approximately one third of the total number migrating from the river each year, and identified 34 individuals (1 individual leaving the river in 2020 was not included) that returned as spawners between 2017 and 2021. This gives an annual average marine survival over the 2015 to 2019 smolt year classes of 1.4%, ranging from 0.4% for the 2015 smolt year class to 2.5% for the 2018 and 2019 smolt year classes (Figure 3, Table S2).

### 3.3 Admixture and Reproductive Success

**FIGURE 4:**
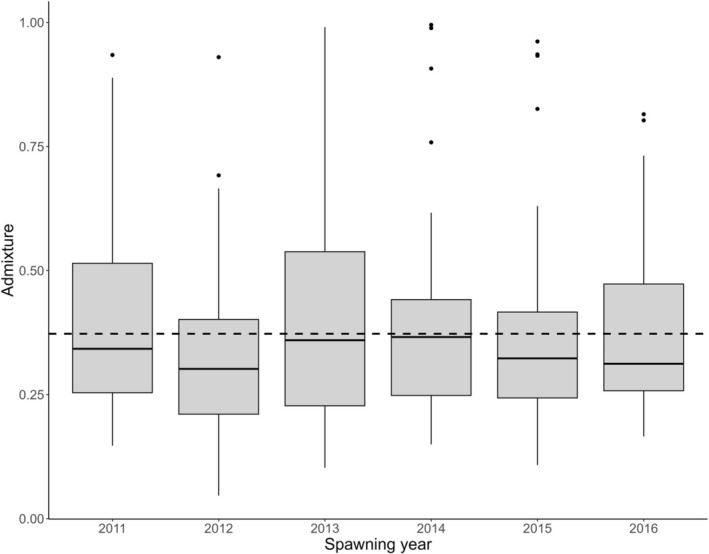
The median and range of admixture estimates for spawners with the overall average admixture shown by the dotted horizontal.

Based on an estimate from genome‐wide SNPs, the average admixture for the spawners entering the river from 2011 to 2016 was computed to be 37% (range: 5%–99%) and was relatively stable between years (Figure 4).

**FIGURE 5:**
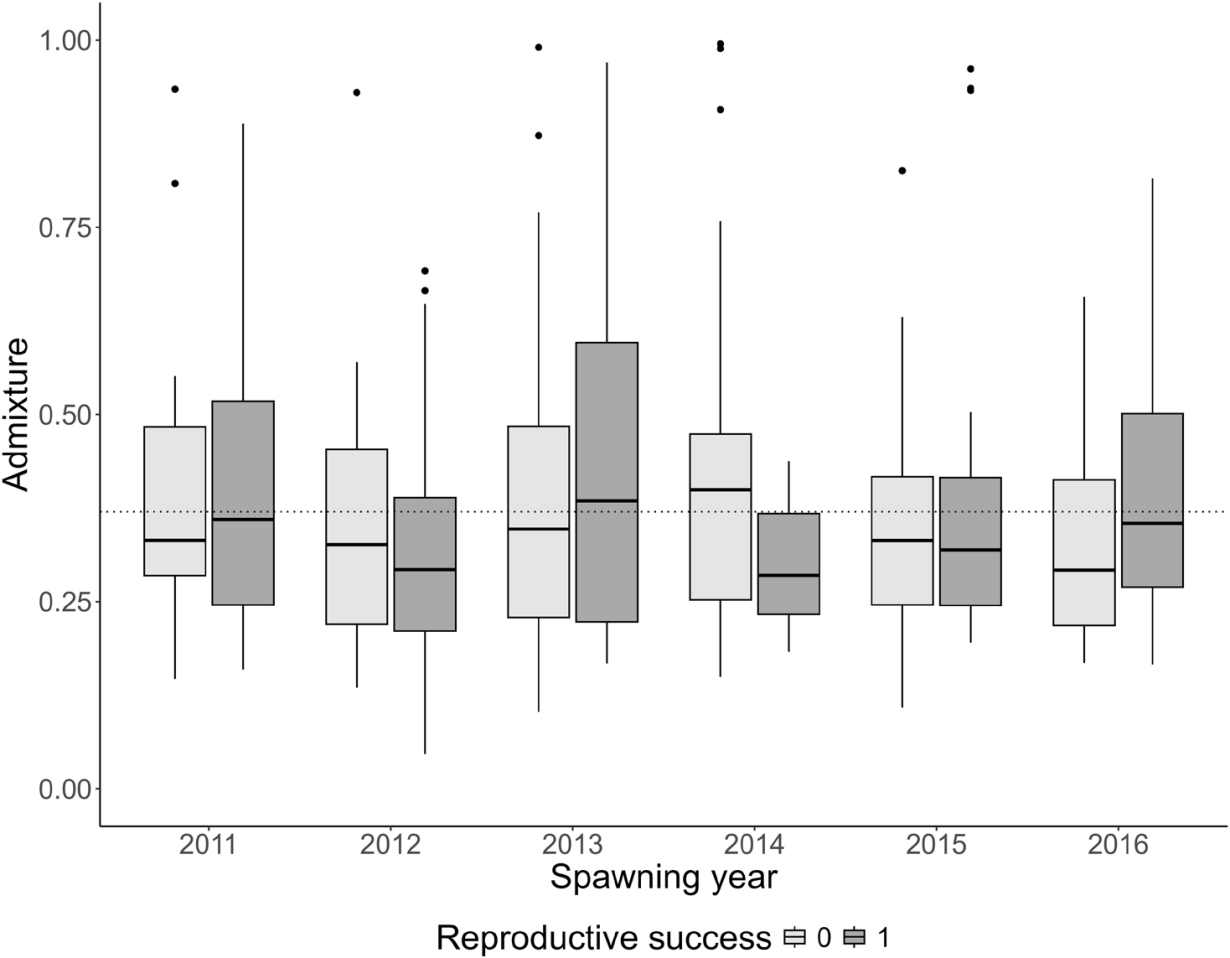
The median and range of admixture estimates for spawners who were assigned (adult success: Yes) and not assigned (adult success: No) as parents to our subsample of smolts. There was no significant influence of admixture on reproductive success over the study period (Table S3).

**FIGURE 6:**
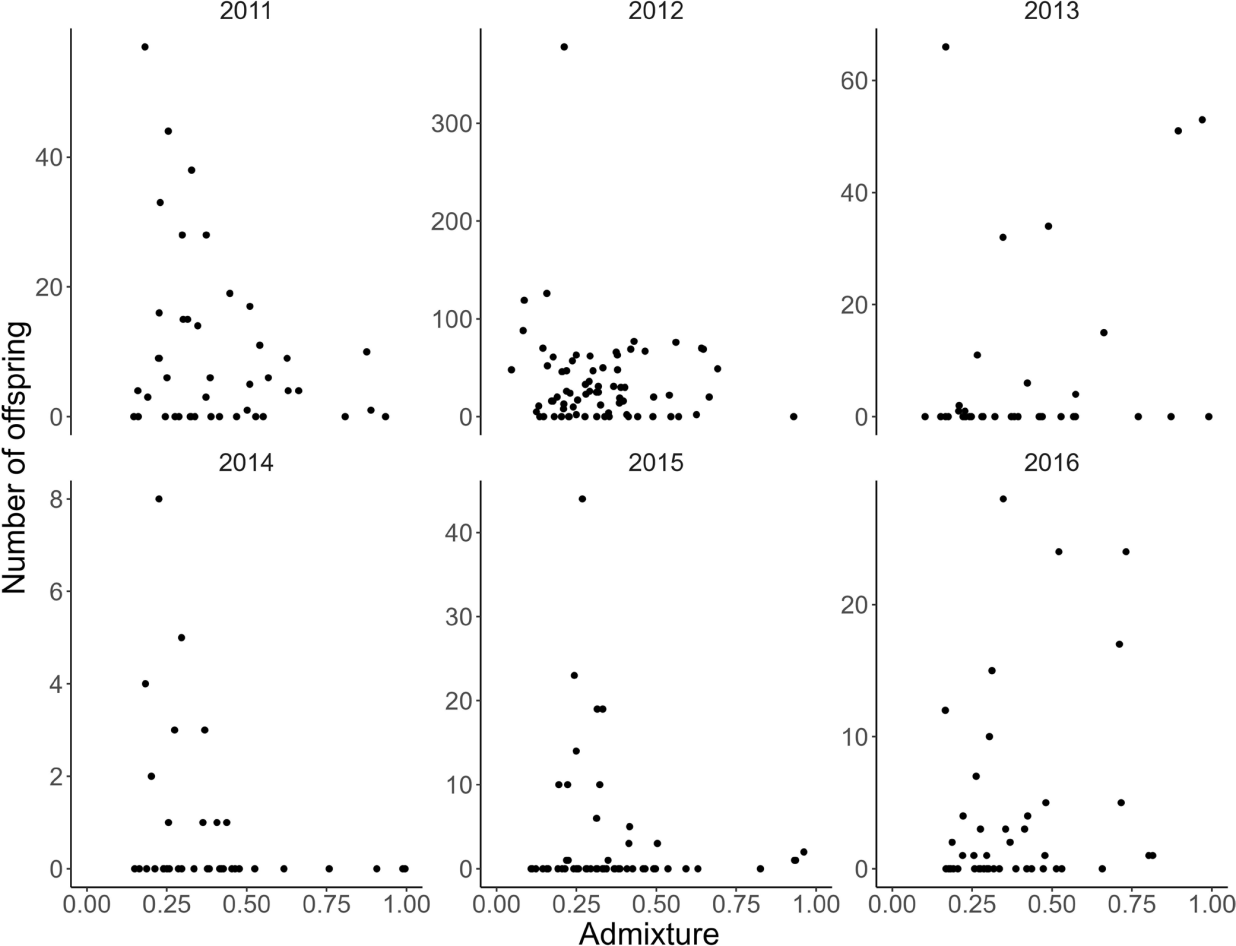
There was no overall significant effect of admixture on the number of assigned smolts per spawner for each spawning year (Table S4).

We successfully assigned 85% of the subsampled smolts back to one or both parental spawners (Figure S4). The smolts lacking one or both parental spawners may come from mature male parr originating from the experimental work conducted before the present study. We thereafter investigated how admixture influenced reproductive success, where success first was defined as whether the adult was assigned as a parent to a smolt, and thereafter defined as the number of smolts produced. We found no significant effect of admixture on being assigned as a parent or not (Figure 5, Table S3), and no significant effect of admixture on the numbers of smolts assigned to a spawner parent when modelled across all spawning years (although there were different trends within some of the years, see Figure 6, Table S4). When modelled as an overall effect, spawner size was not significantly associated with individual admixture (Figure S5, Table S5).

We found no significant difference between the average spawner admixture (37%, 291 individuals) and the estimated admixture of the offspring recruited back to the river (34%, 18 individuals with both parents assigned to them) (*t* = −1.00, df = 24, *p* = 0.325).

## 4 Discussion

We observed that the levels of domestication‐admixture among straying salmon that colonised the river Guddal did not affect their relative reproductive success, as estimated by their assignment as parents and the total number of smolts attributed to each parent over the years. We also observed that the freshwater and marine productivity levels of the resulting population are within those observed in other wild salmon populations, despite the average admixture of the population being regarded as high (37%). On average over one‐third of the genome of an individual is of domesticated genetic ancestry in the population, which would indicate a very poor genetic condition (more than 10% admixture) of the population based on the classification system used in a national report series that up until now has assessed admixture levels in 250 wild salmon populations in Norway (Diserud et al. 2023). The present study shows, to our knowledge for the first time, that a high level of domestication‐admixture does not preclude a productive population trajectory. These data thus have significant implications for how we view the long‐term consequences of domestication‐admixture in wild salmonid populations and the role of natural selection in purging unwanted genotypes over generations.

Upon initial inspection, the results of the current study may be regarded as somewhat unexpected based on previous studies that demonstrate lower fitness of domesticated individuals or their offspring in the wild. Fleming et al. (2000) observed that mature adult domesticated salmon released within a river containing a wild salmon population had a lower breeding success compared to the wild population and that their offspring displayed lower survival in comparison with the offspring of wild salmon during the early freshwater phase. McGinnity et al. (1997) found that freshwater survival of domesticated salmon planted out in a river as eyed eggs was lower compared to the wild salmon originating from the river, also planted out as eyed eggs. Similarly, Skaala et al. (2019) found that domesticated salmon had lower egg to adult survival versus a non‐local wild strain. However, it is important to note that the present study does not compare the reproductive ability of admixed to pure wild salmon; therefore, it is not directly comparable to the above. Several of the studies also use partial artificial rearing in their experiments, with the planting of eyed eggs or the release of fish reared for a part of their life cycle in a hatchery environment. Here, we have compared the reproductive success between naturally spawning individuals with varying admixture levels within the same river population, where we observed no effect of admixture on reproductive success.

As there was no wild salmon population present in the river Guddal prior to 2011, we believe that the near absence of intra‐specific competition should be considered when interpreting our results. The presence of a native salmon population would buffer against the so‐called genetic swamping of an inflow of maladapted genes and likely modulate the relative success of the admixed strayers entering the river Guddal. Several studies suggest that wild population density is a determinant of the relative success of escaped farmed salmon (Glover et al. 2012; Vollset et al. 2014; Mahlum et al. 2021; Diserud et al. 2022). Glover et al. (2012) investigated temporal genetic changes in 21 salmon populations and compared the level of introgression estimated for each population to the level of escaped farmed salmon observed on their respective spawning ground. They found that populations with lower densities of adult wild spawners had higher levels of introgression detected than populations with high densities of adult spawners, concluding that the probability for introgression increased with decreased wild spawner density and thus competition (Glover et al. 2012). Thus, when densities of wild spawners are low, as in the present study, an absence of competition from local populations may facilitate colonisation for escaped domesticated fish or strayers, potentially with an among‐population genetic homogenising effect (Skaala et al. 2006).

We observed that freshwater productivity in the river Guddal was within the range of native salmon populations from similar smaller rivers in Norway and Scotland, where comparable estimates were available. For Norway there are two other rivers with smolt trapping facilities, the river Imsa and the river Dale, where smolt production estimates are 6–7 smolts per 100 m^2^. In both cases, the local population is supplemented with hatchery fish, and therefore the estimates of smolt production for these rivers are not entirely based on naturally recruited wild populations. Two small Scottish rivers with smolt traps also have data on smolt productivity (Glover and Malcolm 2015 (first published)). The trap‐based smolt production estimates for these four rivers were 2–7 smolts per 100 m^2^. Smolt production estimates from the first‐generation colonisation of Guddal by highly domestication‐admixed strayers, that is, ~4 smolts per 100 m^2^, are thus within these ranges (Table S6).

The computed marine survival for the fish produced in the river Guddal falls within the lower range of marine survival estimates for a similar time period for rivers with native salmon populations in the Northeastern Atlantic (Figure 3) (ICES 2020, 2024). The area in which the river Guddal is located, the Hardangerfjord, is one of the most salmon‐farming intensive regions in Norway. Vollset et al. (2014) found that rivers located further in the Hardangerfjord contained lower densities of adult salmon than rivers located in the outer part of the fjord. The authors suggest that a higher cumulative mortality for rivers with longer migration distances could at least in part explain this observation. Similarly, Utne and Mousing (2025) found that simulated post‐smolts migrating from rivers in western Norway needed to first migrate through oceanic areas of potential low prey abundance, while post‐smolts from rivers in middle Norway have a more direct route to prey‐rich feeding grounds. The lower marine survival observed in the present study in the river Guddal may therefore be due to the challenging migration route out of the fjord to oceanic feeding grounds. Most of the rivers used in the present study to compare marine survival have a much more direct route to the ocean than the river Guddal. We also observed no evidence for selection against admixture for those individuals with both assigned parent spawners that were recruited back to the population, where the average admixture of those recruited back to the population was 34% (offspring admixture taken as an average of the parent's admixture).

Individual admixture estimates observed among the colonising adults in the present study ranged from 0.05 to 0.99, with an average of 37%. This suggests that they result from admixture events over several generations back in time, as witnessed by the large number of escapees observed in rivers in this region since the late 1980's (Diserud et al. 2019). For example, assuming a generation time of 5–6 years (Hutchings and Jones 1998; Jensen et al. 2022), the most direct explanation of the presence of individuals with an admixture estimate of 0.5 is an escaped farmed salmon crossing with a wild salmon one generation prior, while an admixture event four generations back in time will result in individuals with an admixture estimate of approximately 0.125. The strayers entering Guddal are thus likely to be the result of up to 5–6 several generations of the agonistic processes of introgression versus natural selection purging mal‐adapted individuals. The rivers Etne and Uskedal, from which the majority of the initial colonisers originate, are documented to contain wild salmon populations that have legacies of domestication‐admixture (Karlsson et al. 2016, Besnier, Ayllon, et al. 2022). Straying is a well‐known phenomenon among salmonid fishes and could be advantageous when it serves to buffer against changes in habitat quality and facilitates recolonisation after local extinctions as well as colonisation of new habitats (Keefer and Caudill 2014). We genetically identified smolts leaving the river that returned in later years to spawn, demonstrating that after the initial colonisation via straying, there is also a self‐recruiting component to the population; however, the high number of strayers continuing to enter the river Guddal suggests that the river functions as a part of a metapopulation of rivers in the Hardangerfjord.

Survival in Atlantic salmon is density‐dependent in the freshwater phase, where the carrying capacity of a river is determined by factors such as available habitat, temperature profile and other biotic and abiotic factors (Jonsson et al. 1998). Survival at sea also varies greatly between populations and is dependent on a number of factors that are density‐independent and make marine mortality hard to predict (Chaput 2012). The 2024 ICES report on the status of Atlantic salmon in the North Atlantic reports return rates for wild smolts in the range of below 1% up to 10% (ICES 2024). Generally, the lifetime success of salmon is therefore low, with few individuals returning to spawn out of hundreds or even thousands of smolts initially leaving a river. The opportunity for selection in all life phases is therefore high, potentially purging maladapted individuals such as the offspring of domesticated and/or admixed fish (Skaala et al. 2012; Wringe et al. 2018; Wacker et al. 2021). For a feral population to successfully colonise a habitat, the population will undergo several genetic, phenotypic and demographic changes that allow the population to adapt to the novel environment (Mabry et al. 2021). Adaptation can occur as quickly as one generation in the wild, given that sufficient genetic variation is present and continued gene flow from the domesticated source is mitigated by natural selection through mortality and competition (Dayan et al. 2024).

The domestication‐admixed strayers entering the river Guddal had already undergone at least one and potentially up to 5 or 6 generations of selection and back‐crossing in the wild, and we therefore speculate that the evolutionary force of natural selection over multiple generations has removed the most mal‐adapted individuals, and that this has played a key role in the colonisation and subsequent productivity of this domesticated‐admixed population. A recent study modelling the effects of domestication‐introgression in wild populations (Glover et al. 2025) found that over time, neutral genetic markers estimated higher admixture levels in a population than markers under selection. This is because neutral genetic markers are gradually dis‐coupled from the adaptive traits through recombination and may linger in the population despite the fact that fitness is gradually re‐instated as markers under selection are purged. Therefore, it is possible that admixture estimates in the present study, and indeed in all other similar studies (Bolstad et al. 2021; Wacker et al. 2021; Besnier, Ayllon, et al. 2022) are to a degree influenced by the degree of selection the loci are under. In turn, this means that as time progresses, the admixture estimates may become more indicative of the past demographic effect of introgression rather than the current biological status. Nevertheless, our admixture estimates are based on the use of 3172 genome‐distributed SNPs and demonstrate that the population recently colonising Guddal displays a high level of domesticated ancestry while simultaneously displaying freshwater and marine productivities overlapping with those observed in locally adapted wild populations.

Our documentation of the timeline of colonisation, combined with accurate productivity and admixture measurements in the spawners that have colonised this river, sheds new knowledge on a globally important topic where it is widely accepted that admixture from domesticated conspecifics will lead to reductions in fitness and productivity of wild populations. We demonstrate that highly admixed fish can rapidly establish a population in the wild, in this specific case likely facilitated by the absence of competition from an existing natural population in the freshwater stage. We further demonstrate that highly admixed fish are capable of successful reproduction and that a population with an overall high estimate of admixture can still be productive, which we speculate is due to the strong effects of natural selection throughout the life cycle of salmon that have purged the most maladapted genes.

## Author Contributions

Conceptualisation: Øystein Skaala, Kevin A. Glover. Funding acquisition: Øystein Skaala. Investigation: Alison C. Harvey, Øystein Skaala, Kevin A. Glover. Methodology: Alison C. Harvey, Francois Besnier, Britt Iren Østebø, Anne Grete Sørvik, Per Tommy Fjeldheim, Laila Unneland. Project administration: Øystein Skaala. Visualisation: Alison C. Harvey. Writing: Alison C. Harvey, Øystein Skaala, Kevin A. Glover, Francois Besnier, Marine S.O. Brieuc, Fernando Ayllon, Kjell R. Utne, Monica F. Solberg.

## Funding

This study was funded by the Nærings‐ og fiskeridepartementet and the Norwegian Environmental Directorate.

## Ethics Statement

Experimental protocol (permit numbers S.nr. 07/13020‐1; S.nr. 2015/33655; S.nr. 17/15858) was approved by the Norwegian Animal Research Authority (NARA). All welfare and use of animals were performed in strict accordance with the Norwegian Animal Welfare Act of 19th June 2009, enforced on the 1st of January 2010. In addition, all personnel involved in the data collection had undergone training approved by the Norwegian Food Safety Authority, which is mandatory for all personnel handling fish.

## Conflicts of Interest

The authors declare no conflicts of interest.

## Supporting information


**Data S1:** ele70319‐sup‐0001‐FigureS1‐S5‐TableS1‐S6.docx.

## Data Availability

All data and code used in the analyses are publicly available at Brage IMR (https://hdl.handle.net/11250/3214122) and in the Dryad Repository DOI: 10.5061/dryad.sn02v6xkm.
