# Supplementary material for: Domestication‐Admixed Atlantic Salmon ( Salmo salar ) Establish a Productive Population in the Wild
Source: Ecol Lett. 2026 Feb 13;29(2):e70319. doi: 10.1111/ele.70319 (PMC12902803; doi:10.1111/ele.70319)
Supplement: Supplementary file 1 — Data S1: ele70319‐sup‐0001‐FigureS1‐S5‐TableS1‐S6.docx. [file ELE-29-0-s001.docx]

Supplementary Material

**Domestication-admixed Atlantic salmon strayers colonise a river in one generation**

Alison C. Harvey*^1^, Øystein Skaala^1^, Francois Besnier^1^, Britt Iren Østebø^1^, Anne Grete Sørvik^1^, Per Tommy Fjeldheim^1^, Laila Unneland^1^, Marine S.O. Brieuc^1^, Fernando Ayllon^1^, Kjell R. Utne^1^, Monica F. Solberg^1^, & Kevin A. Glover^1^

Corresponding author: alison.harvey@hi.no.

MATURE ADULTS

MATURE PARR

SMOLTS

EGGS

ALEVINS

FRY

PARR

FEEDING

ADULTS

FRESHWATER

1-6 years

SEAWATER

1-5 years

SPAWNING

Figure S1: Simplified life cycle of the anadromous Atlantic salmon.


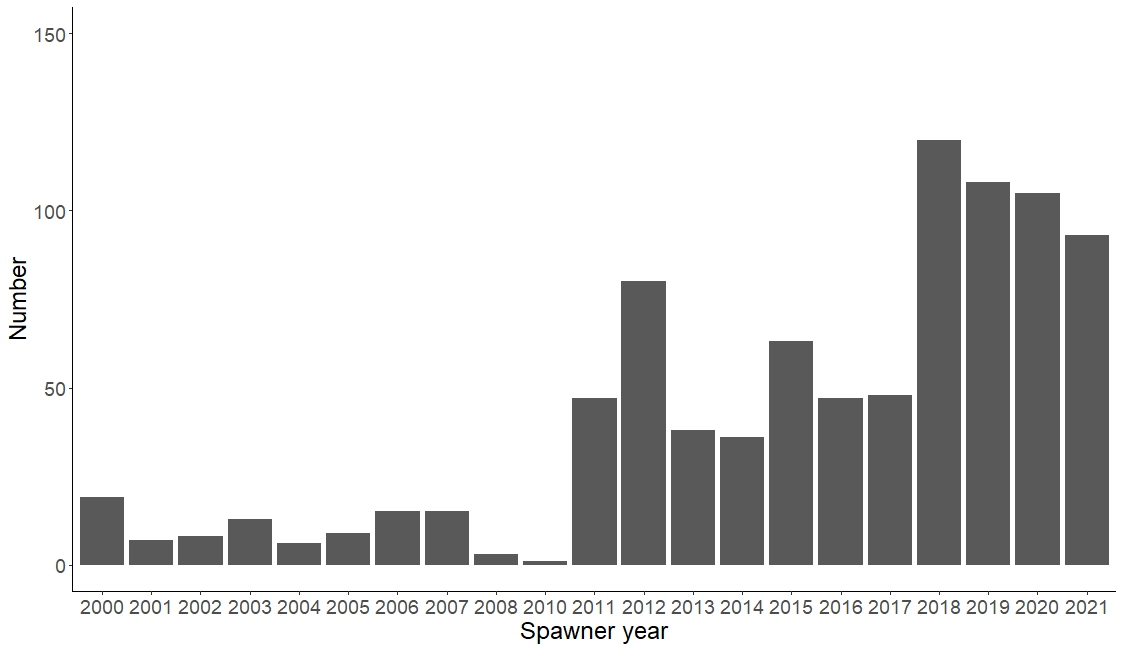


Figure S2: the numbers of spawners entering the trap each year from 2000 to 2021.


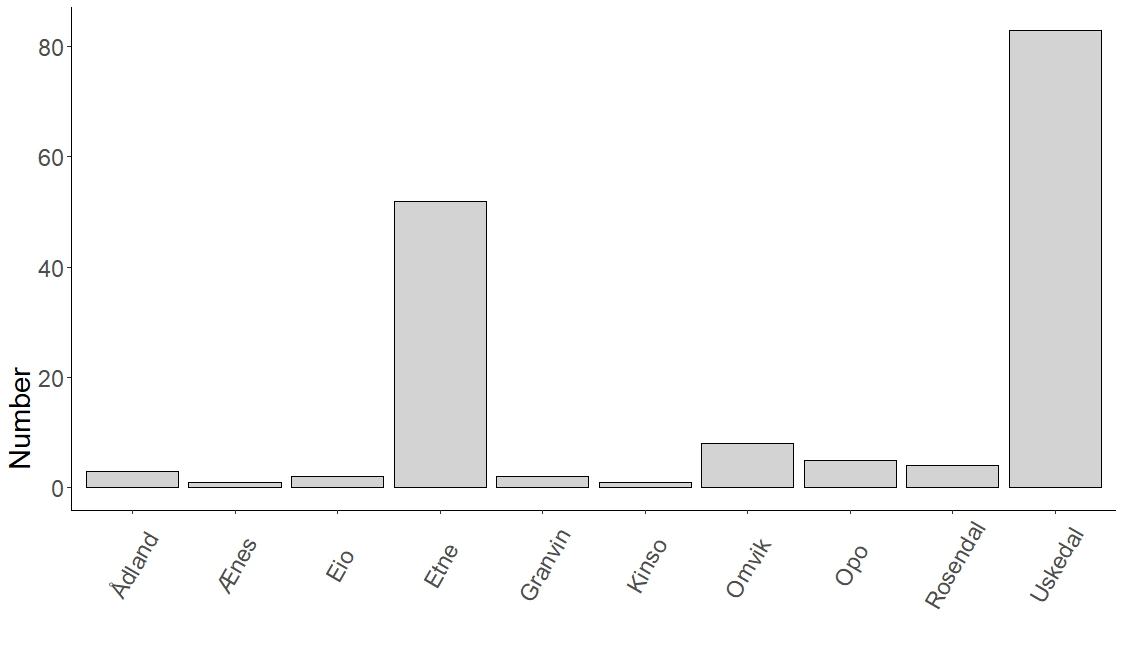


Figure S3: The number of spawners entering the river Guddal between 2011-2016 that were genetically assigned to each river in the baseline with a probability of above 0.80.


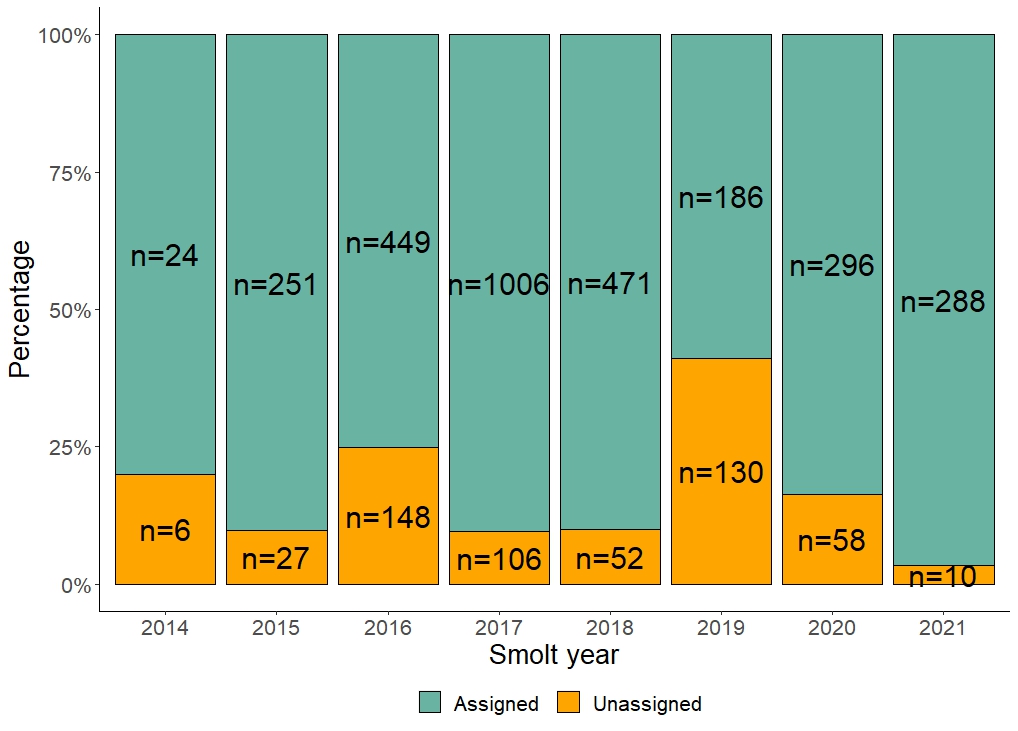


Figure S4: The number of smolts that were successfully assigned to one or more parent or not assigned to any parent for each smolt year class.


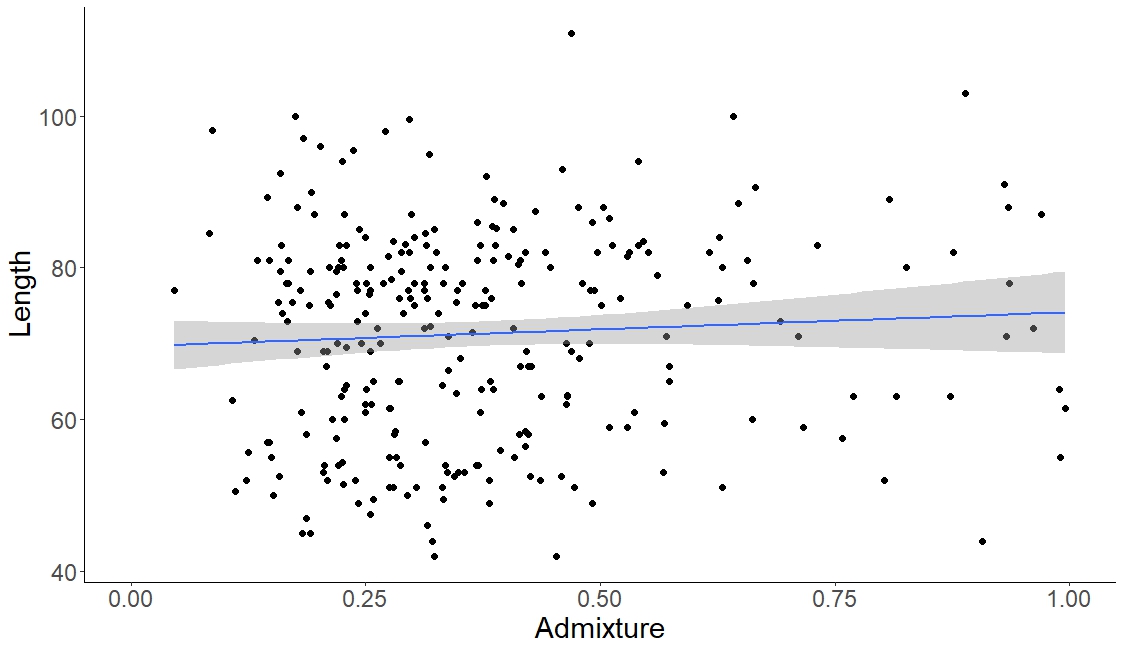


Figure S5: The influence of spawner size (length in centimeters) on admixture. The influence of size on admixture was positive.

Table S1: Calculated smolt production (smolts/100m^2^) for smolt years 2015 to 2021. Any smolts pertaining to the field experiments or supplementary egg planting have been accounted for and removed from the dataset.

| Year | N smolts (all) | Area 41 578 m2 |
| --- | --- | --- |
| 2015 | 878 | 2.1 |
| 2016 | 1987 | 4.8 |
| 2017 | 3514 | 8.5 |
| 2018 | 1109 | 2.7 |
| 2019 | 1297 | 3.1 |
| 2020 | 1755 | 4.2 |
| 2021 | 1091 | 2.6 |
| Average | 1662 | 4.0 |
|  |  |  |

Table S2: Calculated marine survival for smolt years 2015 to 2019.Any smolts pertaining to the field experiments or supplementary egg planting have been accounted for and removed from the dataset.

|  | N smolts | N genotyped smolts | N returning as spawners | Marine survival % |
| --- | --- | --- | --- | --- |
| 2015 | 878 | 278 | 1 | 0.36 % |
| 2016 | 1987 | 597 | 7 | 1.17 % |
| 2017 | 3514 | 1112 | 5 | 0.45 % |
| 2018 | 1109 | 523 | 13 | 2.49 % |
| 2019 | 1297 | 316 | 8 | 2.53 % |
| Summary | 8785 | 2826 | 34 | 1.20 % |

Table S3: Summary results for the models investigating the influence of admixture and sex on spawning success.

| Fixed effects |  |  |  |  |
| --- | --- | --- | --- | --- |
|  | Estimate/Beta | SE | Z | P value |
| Intercept | 0.314 | 0.488 | 0.643 | 0.520 |
| Admixture | -0.215 | 0.941 | -0.228 | 0.819 |
| Sex | -1.066 | 0.559 | -1.907 | 0.057 |
|  |  |  |  |  |
| Admixture x Sex | 1.213 | 1.309 | 0.927 | 0.354 |
| Random Effects |  |  |  |  |
|  |  | Variance | SD | Correlation |
| Year (Intercept) |  | 0.502 | 0.709 |  |

Table S4: Summary results for the models investigating the influence of admixture, spawning year and sex on number of offspring (smolts) produced by the spawners.

| Fixed effects |  |  |  |  |
| --- | --- | --- | --- | --- |
|  | Estimate/Beta | SE | Z | P value |
| Intercept | 2.859 | 0.251 | 11.387 | 0.000 |
| Admixture | -0.417 | 0.484 | -0.862 | 0.389 |
| Sex | 0.059 | 0.332 | 0.177 | 0.860 |
| Year 2012 | 0.950 | 0.207 | 4.590 | 0.000 |
| Year 2013 | 0.575 | 0.328 | 1.754 | 0.080 |
| Year 2014 | -1.666 | 0.374 | -4.458 | 0.000 |
| Year 2015 | -0.443 | 0.276 | -1.605 | 0.109 |
| Year 2016 | -0.616 | 0.263 | -2.339 | 0.019 |
|  |  |  |  |  |
| Admixture x Sex | -0.298 | 0.772 | -0.386 | 0.700 |
| Random Effects |  |  |  |  |

Table S5: Summary results for the models investigating the influence of size and sex on individual admixture of the spawners.

| Fixed effects |  |  |  |  |
| --- | --- | --- | --- | --- |
|  | Estimate/Beta | SE | Z | P value |
| Intercept | -1.799 | 0.352 | -5.116 | 0.000 |
| Length | 0.010 | 0.004 | 2.271 | 0.023 |
| Sex | 0.454 | 0.397 | 1.142 | 0.254 |
|  |  |  |  |  |
| Length x Sex | -0.004 | 0.005 | -0.798 | 0.425 |
| Random Effects |  |  |  |  |
|  |  | Variance | SD | Correlation |
| Year (Intercept) |  | 0.004 | 0.063 |  |

Table S6: Calculated smolt production (smolts/100m^2^) for rivers with trap data from recent years.

| River | Years | Average N smolts | Area | Smolts/100m^2^ |
| --- | --- | --- | --- | --- |
| Ims (Norway) | 2015-2020 | 602 | 10 000 | 6.0 |
| Dale (Norway) | 2015-2020 | 2205 | 32 120 | 6.9 |
| Girnock (Scotland) | 2015-2021 | 1796 | 74 107 | 2.4 |
| Baddoch (Scotland) | 2015-2021 | 1740 | 37 804 | 4.6 |
